# Effect of soil conditioning on the permeability of coarse-grained soil in mechanized tunnelling

**DOI:** 10.1016/j.heliyon.2023.e22640

**Published:** 2023-11-25

**Authors:** Farjam Salmanpour, Hamid Chakeri, Sajjad Chehreghani, Hossein Azad Soula

**Affiliations:** a Department of Mining Engineering, Sahand University of Technology, Tabriz, Iran; b Department of Mining Engineering, Faculty of Engineering, Urmia University, Urmia, Iran

**Keywords:** Soil conditioning, Foam, Permeability, FIR, FER, Coarse-grained soil

## Abstract

In coarse-grained soils, the reduction of soil permeability via conditioning can effectively prevent groundwater from entering the chamber, thus providing better tunnel face control and ultimately preventing excessive settlements of the surface. In this study, several mixtures of coarse-grained and soil-foam mixtures were utilized in experiments. In which, effects of the foam expansion ratio, foam injection ratio, soil water content, pressure, and grain size distribution on the soil permeability were investigated. In these experiments, two soil types of poorly graded sand (SP) and poorly graded sand with silt and gravel (SP-SM), with different grain sizes were utilized. Based on the experimental results, it has been observed that the soil permeability increases with increasing foam expansion ratio, water pressure, and the coarse-grained portion of the soil. Meanwhile, soil permeability decreases with increasing foam injection ratio and soil water content. Based on the observations, it can be inferred that optimal soil permeability for soil moisture of 15 %, the pressure of 1 bar with a certain grain size occurs at a foam expansion ratio of 7.5 and a foam injection ratio of 52 %.

## 1 Introduction

In tunnel excavations conducted by earth pressure balance tunnel boring machines (EPB-TBMs), the tunnel face can be stabilized by placing the excavated soil in the chamber and balancing the soil pressure by reducing the soil permeability [[1], [2], [3], [4], [5], [6], [7]]. The aforementioned soil must have specific characteristics to create such conditions. When groundwater is present, these characteristics include suitable plastic behavior, low shear strength, high compressibility, and low permeability [[8], [9], [10], [11]]. A geotechnical and surface risk of tunnel excavation with TBM in difficult ground conditions was conducted by several researchers with numerical and experimental modeling [[12], [13], [14], [15], [16], [17]]. In such conditions, during excavation, the tunnel face will remain stable for up to several hours. Water drainage in the mentioned conditions can cause unstable forces in the soil mass. In highly permeable ground (e.g. k = 10-2 m/s), face instability will occur within a few minutes. A permeability parameter equal to 1*10-5 m/s is acceptable to prevent these instabilities and thus eliminate the destabilizing forces [18]. Reaching this permeability level can be achieved by reducing and increasing the amount of additives.

The standard test method for permeability, as recommended in ASTM D2434 and CEN ISO/TS 17892-11, requires a constant water flow throughout testing, which is not acceptable for conditioned soils [19]. This is because soil conditioning agents, such as foam bubbles are washed away and destroyed by water, therefore, the permeability of conditioned soils is not measured [2]. To resolve this issue, some experiments were carried out to measure the conditioned soil permeability. Some researchers have studied the permeability properties of conditioned sandy soils with foam. **Quebaud et al. (1998)** and **Qiao (2009)** tested conditioned soil permeability under different conditions. They found that the permeability coefficient of conditioned soils decreases with increasing the foam injection ratio (FIR) [20,21]. In addition, **Quebaud et al. (1998)** conducted a permeability test on three soil samples with different grain sizes [19]. The first soil sample was siliceous sand without silt with a grain size range from 200 to 400 μm (S1). The second soil sample was siliceous sand with a grain size range from 0 to 4 mm, containing more silts than S1 (S2). The third soil sample was a siliceous aggregate with a grain size range from 4 to 20 mm, (G1). This study found that both very coarse-grained and very fine-grained soils could not be well conditioned with foam; the permeability coefficient can only be significantly reduced if the soil is medium grain-sized.

**Bezuijen et al. (1999)** studied conditioned soil permeability associated with foam filling. By adding foam fills in the soil pores, the conditioned soil's permeability was reduced [22]. **Borio and Peila (2010)** and **Peila (2014)**, by providing the Ix index, showed that as the soil grains become finer, the conditioned soil's permeability decreases [2,3]. **Budach and Thewes (2015)** conducted permeability tests on nine types of soils under different conditions, including 10 % of moisture content and FIR between zero to 65 %. They found that the reduction of the soil permeability induced by foam was more pronounced for sandy soil than for the coarse-grained soil sample [8]. **Kim et al. (2019)** found that adding foam can greatly reduce soil permeability in different conditions [23]. **Huang et al. (2019)** examined the soil grading effect on permeability, and found that the effective particle size (d10) could significantly affect soil permeability characteristics, especially the initial permeability coefficient [24].

**Zhou and Yang (2020)** studied the effect of foam parameters on the permeability of cohesionless soils and their application to prevent water seepage. The results of permeability experiments and field tests indicated highly stable foaming agents with a moderate FER (=10–20 %) and FIR (=50%–75 %) as an effective means to reduce the permeability of cohesionless soils and prevent water seepage from screw conveyors [25].

**Hu et al. (2020)** investigated the effect of hydraulic gradient on the permeability of sand that has been conditioned with foam in various conditioning states. The results show that with an increase in the hydraulic gradient, the range of conditioning parameters of the soil that satisfies the shield tunnelling permeability requirement gradually narrows and moves towards a low water content (w) and high foam injection ratio (FIR) [26].

Based on the available literature, it is clear that the simultaneous investigation of the effect of pressure and foam parameters on permeability in coarse-grained soils is very limited. The focus of the present research is to evaluate the effect of the foam expansion ratio (FER), foam injection ratio (FIR), soil water content, pressure, and grain size distribution on soil permeability and soil conditioning in tunnel excavation with TBM. In these experiments, two types of poorly graded sand (SP) and poorly graded sand with silt and gravel (SP-SM), with different grain sizes were utilized (specify the grain sizes). Based on the experimental results, the soil permeability increases with increasing FER and pressure, and decreases with increasing FIR and soil water content.

## 2 Methods and materials

**Fig. 1:**
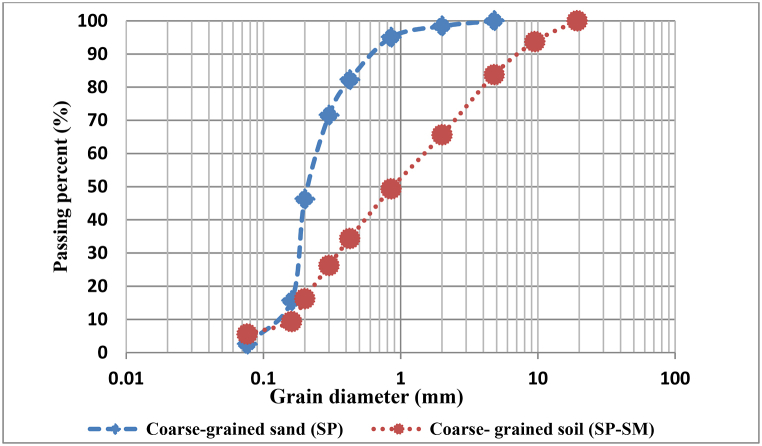
Grain size distribution curve.

The permeability tests were performed by standard permeability devices using conditioned soil samples at FIRs of 40, 50, 60, and 70 %, and FERs of 7.5 and 12. These values were selected based on various implemented projects, such as the Tabriz Metro Lines and the EFNARC recommendations [27]. A constant hydraulic pressure was applied. The grain size distribution curve of the soils used in the experiments is shown in Fig. 1.

**Fig. 2:**
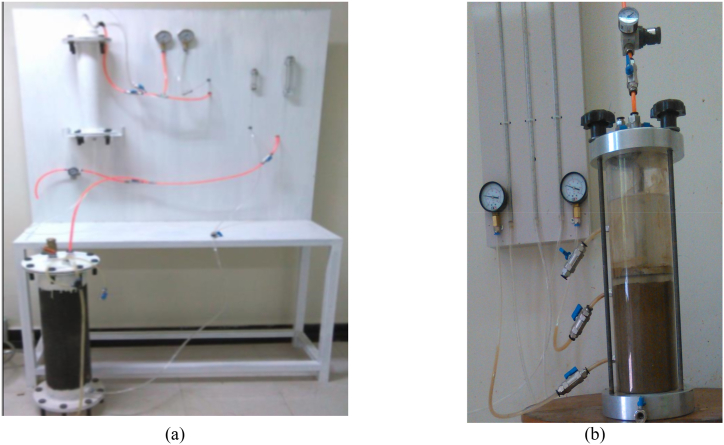
a) Foam generator, b) Modified permeability test.

The standard amount of water passing through the specimens was considered to be 2 L. The method and steps of the tests are as follows:

To improve the soil conditioning, the foam was prepared using a foam generator (Fig. 2(a)) at different FERs. The correct amount of foam was mixed with the soil sample using a mixer so that the foam was completely absorbed by the soil. The resulting mixture was poured into the cylinder of the permeability test device and compacted based on the proctor compaction test [28]. Finally, the cylinder cap was closed, and the sample was subjected to constant water pressure. The permeability was then calculated by analyzing the water flow through the specimen. The experimentation procedure can be observed in Fig. 2(b).

Soil water contents of 10 and 15 % were considered, and the tests were performed with FER values of 7.5 and 12, and FIR values of 40, 50, 60, and 70 %. To examine the effect of pressure application on the duration of water passage through the conditioned soil, tests were conducted under pressures of 0.5, 1, and 1.5 bar. In addition, to investigate the effect of grain size distribution on soil permeability, experiments were carried out on coarse-grained sand with an FIR of 40 %, FER of 7.5 and 12, and soil moisture of 10 %.

## 3 Results

The permeability tests were conducted at pressures of 0.5, 1, and 1.5 bar and soil moisture contents of 10 and 15 %. The effects of soil moisture content and pressure on the permeability of the soil conditioned with foam and with different percentages of FER and FIR were studied.

**Fig. 3:**
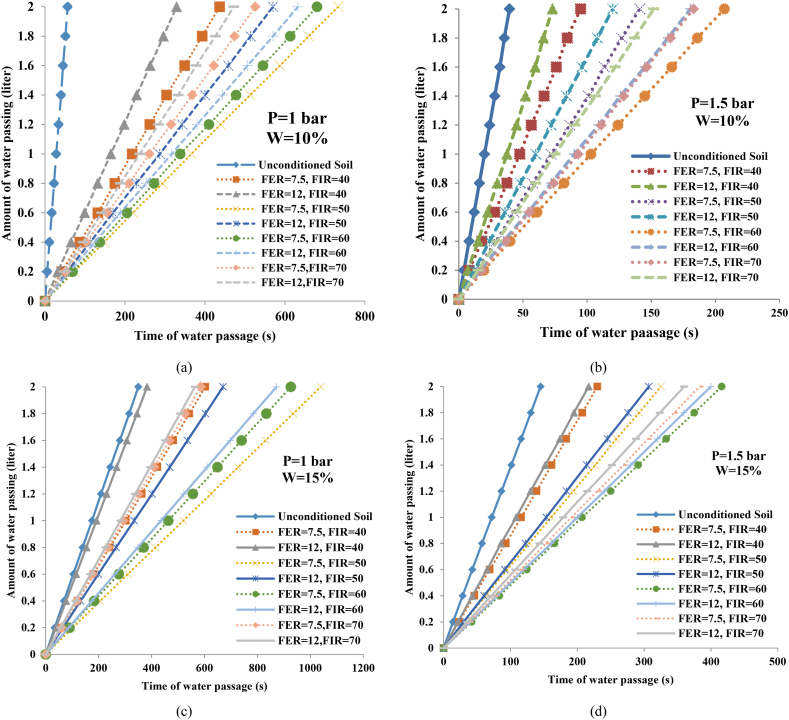
Water passage time versus the volume of water passing for different FERs and FIRs (SP-SM): (a) P = 1 bar/W = 10 %; (b) P = 1.5 bar/W = 10 %; (c) P = 1 bar/W = 15 %; (d) P = 1.5 bar/W = 15 %.

**Fig. 4:**
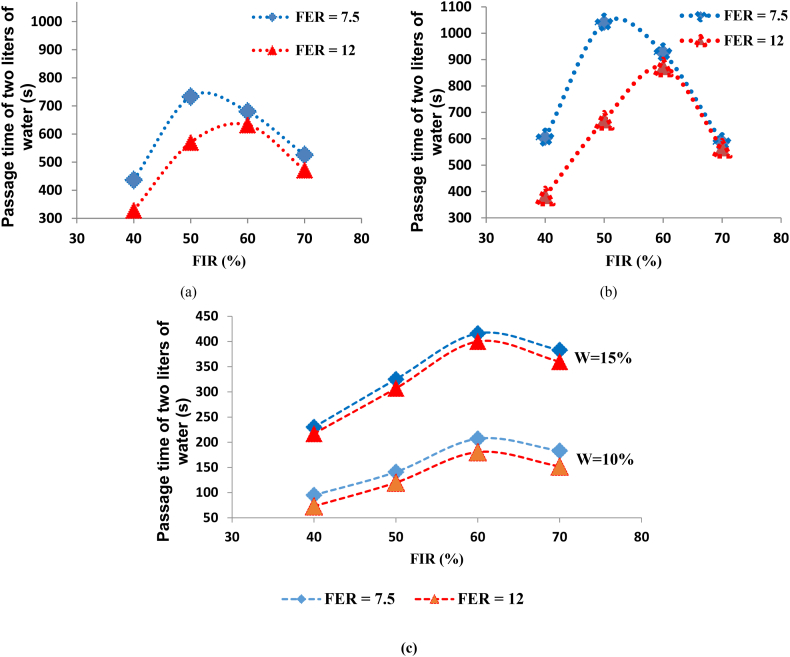
Passage time of 2 L of water versus FIR for different FERs: (a) P = 1 bar and W = 10 %; (b) P = 1 bar and W = 15 %; (c) P = 1.5 bar.

Fig. 3 shows the water passage time diagram (horizontal axis) versus the water volume passing through the sample (vertical axis) for different FERs and FIRs. The effects of the FIR and FER parameters were also investigated for different cases, such as pressures of 1 and 1.5 bar and soil moisture of 10 and 15 %. In addition, Fig. 4 shows the water passage time (2 L) through the sample (vertical axis) relative to the FIR (horizontal axis) for different FERs.

### 3.1 The effect of FER and FIR on permeability

As presented in Fig. 3, the injection of foam for each FER and FIR increased the water passage time compared to the unconditioned soil. In Fig. 3(a), the pressure was kept at a constant 1 bar, and the soil moisture was at 10 %. According to this diagram, by increasing the FER, the water passage time through the sample decreases, and as a result, the permeability increases. For example, in the conditioned soil with an FIR of 40 %, the passage time of 2 L of water for an FER of 7.5 was equal to 436 s, and it was 329 s for an FER of 12. On the other hand, by increasing the FIR, the water passage time decreased, and consequently the permeability decreased. This is true for soils with FIRs lower than 50 % at an FER of 7.5, and FIRs lower than 60 % at an FER of 12. Should the foam injection be higher than the mentioned values, the water passage time decreases and the permeability increases. This decrease is observed in the conditioned soils with an FER of 7.5 and FIR of 60 and 70 %, and also in soils with an FER of 12 and FIR of 70 %.

According to Fig. 4(a), at the pressure of 1 bar and moisture content of 10 %, the conditioned soil with an FER of 7.5 and FIR of 50 % had the lowest permeability. In these conditions, the passage time of 2 L of water through the sample was equal to 733 s. Also, the conditioned soil with an FER of 12 and an FIR of 40 % had a higher permeability. In Fig. 3(b) and Fig. 4(c), the soil moisture was equal to 10 %, and the pressure is was increased to 1.5 bar. In this case, as FER increased, the water passage time through the sample decreased. For instance, in the case of conditioned soil with an FIR of 40 %, the passage time of 2 L of water for an FER of 7.5 was equal to 95 s, and it was 73 s for an FER of 12. The values obtained in this case are smaller compared to the previous case, which shows the effect of pressure.

According to Fig. 4(c), at a pressure of 1.5 bar and a moisture content of 10 %, the conditioned soil with an FER of 7.5 and FIR of 60 % and the conditioned soil with an FER of 12 and FIR of 40 % had low and high permeability, respectively. Additionally, the passage times of 2 L of water in these cases were 207 and 73 s, respectively. Then, soil with 15 % moisture was used, and the pressure was maintained at 1 bar (Fig. 3(c)). According to Fig. 4(b), the trend was similar to the first case; for conditioned soil with an FIR of 40 %, the passage time of 2 L of water for an FER of 7.5 was equal to 603 s, and it was 383 s for an FER of 12.

According to the data provided in these figures, the water passage time for the unconditioned soil is very low compared to the conditioned soils. However, in this case, it can be observed that under the same conditions, the conditioned soil with an FIR of 40 % and FER of 12 had a water passage time close to the unconditioned soil sample, which represents the weakest yield for soil conditioning (Fig. 3(c)). In this case, the conditioned soil with an FER of 7.5 and FIR of 50 % had the lower permeability. Additionally, the passage time of 2 L of water from the sample in this condition was equal to 1039 s. Also, the conditioned soil with an FER of 12 and an FIR of 40 % was found to have a higher permeability, so the passage time of 2 L of water was equal to 383 s.

In the final case, a soil moisture of 15 % was considered, and the pressure was increased to 1.5 bar (Fig. 3(d)). This case followed the same trend as the second case (Fig. 4(c)). The results showed that the conditioned soil with an FER of 7.5 and an FIR of 60 % and the conditioned soil with an FER of 12 and an FIR of 40 % have lower and higher permeability, respectively. Furthermore, the passage time of 2 L of water in these cases is 416 and 217 s, respectively. The result indicate that the pressure in the permeability of both soils rapidly increased when the FIR was increased from 40 to 60.

### 3.2 Effect of soil moisture on permeability

The soil water content was altered from 10 to 15 % to determine its effect while keeping the other parameters, such as the pressure, FIR, and FER, constant during the test. According to the results, when increasing the soil water content, the time of water passing through the conditioned soil increases, and as a result, the permeability of the soil-foam mixture decreases (Fig. 3). It can be stated that, as the moisture in the soil increase, the particles inside the soil become more mobile, filling the pores and discontinuities, thus preventing the flow of water and reducing permeability. For example, when the pressure, FER, and FIR are equal to 1 bar, 7.5, and 50 %, respectively, the time it takes for 2 L of water to pass through the soil with 10 % moisture was equal to 733 s, and for a conditioned soil with 15 % moisture was equal to 1039 s.

### 3.3 Effect of pressure on the permeability

To evaluate the effect of pressure on the permeability of the conditioned soil, other effective parameters were kept constant. To this end, pressures of 0.5, 1, and 1.5 bar were applied to the soil samples. However, it should be noted that in the experiment with a pressure of 0.5 bar after several hours, the water did not flow from the soil-foam mixture. Therefore, the sample is considered entirely impermeable in this case. Finally, comparing the results, it was observed that with increasing pressure, the water passage time through the conditioned soil decreased, and as a result, the permeability increased (Fig. 3).

According to these results, the higher water passage time is related to the samples in which the pressure is equal to 1 bar. It was observed that the permeability at a pressure of 1.5 bar was higher in comparison to 1 bar, indicating the high influence of pressure on permeability. By comparing the graphs in Fig. 4, it was found that in conditioned soils with moistures of 15 % compared to 10 %, the pressure increase had a small effect on increasing the water passage time. Therefore, it can be stated that when decreasing the soil moisture, the pressure effect on permeability increases, and vice versa.

### 3.4 Effect of grain size distribution on permeability

**Fig. 5:**
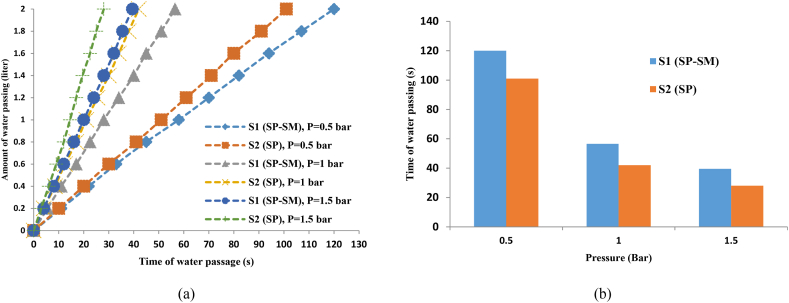
**a)** Water passage time versus amount of water passing for different conditions (unconditioned soils) b) Passage time of 2 L of water in various pressures for unconditioned soils.

According to Fig. 5 (a, b) and the diagram of the grain size distribution (Fig. 1), it was observed that the water passage time through unconditioned soils due to the coarse grain size of poorly graded sand with silt (SP-SM: S1 soil) was lower than poorly graded sand (SP: S2 soil). In other words, as the soil becomes coarse-grained, the water passage time decreases, resulting in an increase in soil permeability.

**Fig. 6:**
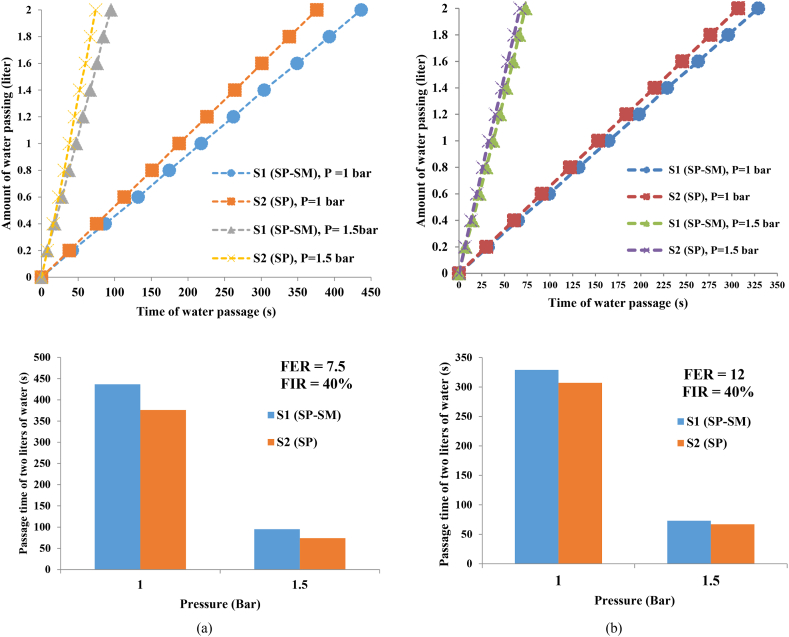
The changes in water passage time relative to changes in the volume of water passing in different conditions for conditioned soil: (a) FER = 7.5 and FIR = 40 %; (b) FER = 12 and FIR = 40 %.

According to the results of the conditioned samples (Fig. 6 (a, b)), it can be observed that after injecting foam in the soil, the water passage time in S1 soil was lower than that of S2 soil. Additionally, the results show that permeability increased as the soil grain size increased, however, this increase was minimal. According to these findings, it can be concluded that after the foam injection, the variation of the permeability of the two types of soil is small.

## 4 Determination of the permeability coefficient for different scenarios

To calculate the permeability coefficient, analog manometers were installed. By installing these manometers, measuring the amount of water head loss inside the sample, and using the permeability formula, the permeability value was calculated. The permeability coefficient is determined by equation (1) in which K is the permeability coefficient (m/s), Δh is the difference of pressures at the sample's top and bottom (m), Q is the flow rate, L is the height of the sample (m), and A is the area of sample cross-section (m^2^).

(1) K=(Q.L)/(A.Δh)

The S2 soil was utilized in these tests, which were performed considering an FER of 7.5, soil moisture of 15 %, and pressures of 1 and 1.5 bars. Also, the FIR for the different tests was considered to be 40, 50, and 70 %. The results are presented in Table 1.

**Table 1:** Permeability coefficient (m/s) for different scenarios.

FIR (%)	FER	Pressure (bar)	Flow rate (m^3^/s)	Permeability coefficient (m/s)
0	7.5	1	3.88*10^−5^	8*10^−4^
0	7.5	1.5	5.97*10^−5^	1.3*10^−3^
40	7.5	1	3.31*10^−6^	2*10^−5^
40	7.5	1.5	8.69*10^−6^	6.5*10^−5^
50	7.5	1	1.92*10^−6^	9.8*10^−6^
50	7.5	1.5	6.15*10^−6^	4.2*10^−5^
70	7.5	1	1.03*10^−6^	5.15*10^−6^
70	7.5	1.5	6.15*10^−6^	3.28*10^−5^
Samples Volume			1.9*10^−3^ m^3^	
Samples Height			20 cm	
Samples Diameter			11 cm	

According to the obtained results (Table 1) and by comparing the soil permeability results of the unconditioned (without foam injection) and conditioned soils, it can be observed that by injecting foam in the soil, the foam penetrates the pores between the soil particles and prevents water from passing through them, which reduces permeability. Based on these results, it can be stated that by increasing the FIR, permeability decreases, while by increasing pressure, permeability increases. All the permeability coefficients presented in this table for the conditioned soil are in the appropriate permeability range (≤10-5 m/s) for drilling with the EPB machine [29]. The permeability coefficient in the conditioned soil with an FIR of 50 % was equal to 9.8*10-6 m/s, which is the ideal permeability value for drilling with an EPB.

## 5 Optimal values of parameters

**Fig. 7:**
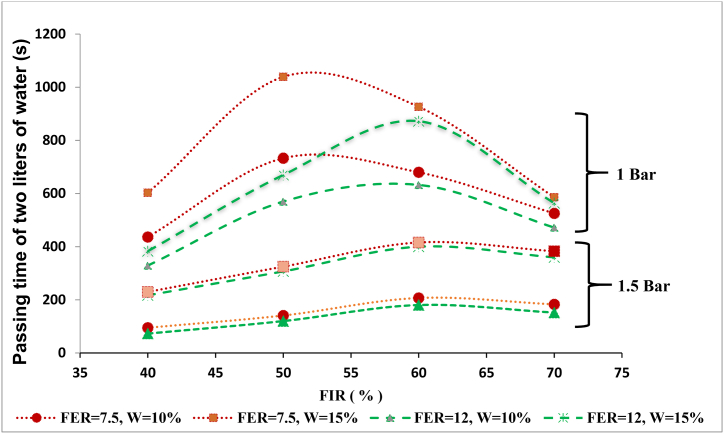
Water passage time versus different FERs, FIRs, moistures, and pressures.

To find the optimal FER and FIR values, the summary of the laboratory results is shown in Fig. 7, and the best interpolation was made for these results. The effects of permeability parameters, including moisture, pressure, FER, and FIR can be observed. In addition, according to this diagram, it is possible to provide the optimal FIR and FER for each moisture value. Based on the results presented in this figure, it can be concluded that lower permeability occurs for a pressure of 1 bar, moisture of 15 %, an FER of 7.5, and an FIR of 52 %. As the FIR increased to more than 52 %, the permeability value increased, and by increasing the FER and pressure, permeability increased.

**Fig. 8:**
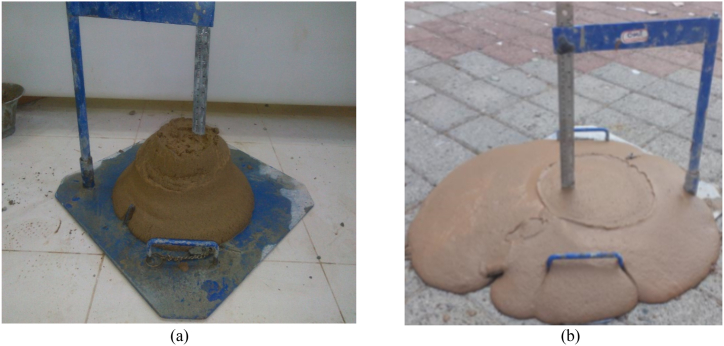
Slump tests for soil and foam mixture: (a) W = 15 %/FER = 7.5/FIR = 52 %; (b) W = 10 %/FER = 7.5/FIR = 61 %.

The slump test is a simple and low cost test for the investigation of conditioned soil properties. The slump tests for two cases of conditioned soils are presented in Fig. 8. Fig. 8(a) illustrates the slump test for the conditioned soil with an FER of 7.5, FIR of 52 %, and moisture of 15 %, while Fig. 8(b) indicates the conditioned soil with an FER of 7.5, FIR of 61 %, and moisture of 10 %. The result show that the slump value increases with the FIR, and when this ratio reaches 61 %, the amount of foam exceeds the capacity of the soil. The suitable range of slump for conditioned S1 soils is between 15 and 20 cm. Therefore, according to the results of the slump tests, an FIR of 52 % can be deemed optimal.

## 6 Conclusion

Tunneling with EPB machines requires a soil conditioning process to facilitate the drilling operations, especially in non-cohesive soils. This process can be conducted by injecting foam, water, or filler materials at the tunnel face, in the chamber, or along the screw conveyor. This study aimed to investigate the effect of foam on the permeability of coarse-grained sands. Therefore, the effects of foam expansion ratio (FER), foam injection ratio (FIR), soil water content, pressure, and grain size distribution on soil permeability were evaluated. To this end, permeability tests were carried out on foam-conditioned soils. The following conclusions can be drawn from the findings of this study:

- As the foam expansion ratio (FER) increases, the water passage time decreases, resulting in an increase in permeability. Furthermore, the time it takes for water to pass through the soil increases and permeability decreases. This trend is valid until the moment that the optimal foam injection ratio is reached. Increasing the FIR to a higher than optimal amount causes an increase in permeability,
- As the percentage of soil moisture increases, the amount of water passing through the soil increases, and as a result, the permeability of the soil-foam mixture decreases,
- As the pressure increases, the permeability of the conditioned soil increases. Pressure has a significant effect. The effect of pressure on permeability is higher than that of the other investigated parameters,
- In the unconditioned state, as the sand becomes coarse-grained, the water passage time decreases, and as a result, the permeability of the soil increases. However, in the conditioned soil state, permeability increases as the sand grain size increases. Nevertheless, the said increase is minimal,
- The permeability coefficient for the conditioned soils with an FIR of 50 % is equal to 9.8*10 -6 m/s, which is the ideal permeability value for drilling with EPB in the sand.

Finally, in order to achieve the optimal FER and FIR values, the effect of all parameters was investigated simultaneously, and it can be concluded that the lower permeability is obtained for a pressure of 1 bar, moisture of 15 %, an FER of 7.5, and an FIR of 52 %.

## Funding

This review has no funding affiliation to declare.

## Data availability statement

Data will be made available on request.

## CRediT authorship contribution statement

**Farjam Salmanpour:** Data curation, Investigation. **Hamid Chakeri:** Investigation, Methodology, Project administration, Writing – original draft. **Sajjad Chehreghani:** Conceptualization, Formal analysis, Investigation, Validation. **Hossein Azad Soula:** Data curation, Resources, Supervision.

## Declaration of competing interest

The authors declare that they have no known competing financial interests or personal relationships that could have appeared to influence the work reported in this paper.
